# The effect of an animation video on consultation time, anxiety and satisfaction in women with abnormal cervical cytology: Animation video reduces colposcopy time

**DOI:** 10.1016/j.pmedr.2019.01.005

**Published:** 2019-01-15

**Authors:** Tirza Wouters, Jenny Soomers, Marieke Smink, Rixt A. Smit, Margreet Plaisier, Saskia Houterman, Ruud L. Bekkers, Angélique A. Schiffer, Victor J. Pop, Jurgen M.J. Piek

**Affiliations:** a Department of Obstetrics and Gynaecology, Catharina Hospital, Eindhoven, the Netherlands; b Department of Obstetrics and Gynaecology, Elizabeth-Tweesteden Hospital, Tilburg, the Netherlands; c Department of Obstetrics and Gynaecology, Jeroen Bosch Hospital, 's Hertogenbosch, the Netherlands; d Department of Education and Research, Catharina Hospital, Eindhoven, the Netherlands; e Department of Psychiatry and Medical Psychology, Catharina Hospital, Eindhoven, the Netherlands; f Department of Medical and Clinical Psychology, Tilburg University, Tilburg, the Netherlands

**Keywords:** GOCZ, Gynaecological Oncology Centre South, PEACE-q, Patient's Experience and Attitude Colposcopy Eindhoven questionnaire, Anxiety, Atypical squamous cells of the cervix, Audiovisual aids, Cervical intraepithelial neoplasia, Colposcopy, Netherlands, Personal satisfaction, Squamous intraepithelial lesions of the cervix

## Abstract

The objective was to assess whether supplementing hospital-dependent standard information with a hospital-independent animation video might reduce consultation time, pre-colposcopy anxiety levels and increase post-colposcopy satisfaction.

Between November 2016 and May 2018, women were included if they were referred to the department of Obstetrics and Gynaecology in one of the three participating hospitals in the Netherlands due to an abnormal cervical smear. Exclusion criteria were colposcopy in the medical history or inability to understand, speak or read Dutch. Two consecutive cohorts were created: a control group that received standard information and an intervention group that received the same plus the animation video. Outcome measures were consultation time, pre-colposcopy anxiety level and post-colposcopy satisfaction. Consultation time was measured using stopwatch. Anxiety was measured using the State-Trait Anxiety Inventory (STAI) and the Hospital Anxiety and Depression Scale (HADS). Satisfaction was measured with the Patient's Experience and Attitude Colposcopy Eindhoven questionnaire (PEACE-q).

In total, 122 women were included, 61 in each group. Baseline characteristics were similar between the two groups. Pre-colposcopy consultation time was significantly reduced in the intervention group (median 140 s) compared to the control group (median 269 s). However, overall consultation time was not reduced. The outcome measures anxiety and satisfaction were not significantly different.

A hospital-independent animation video did significantly reduced pre-colposcopy consultation time but did not reduce anxiety or increase satisfaction in women with abnormal cervical cytology. Further research should focus on the effects of animation video in a primary care setting.

## 1 Introduction

Previous studies show that referral to a gynaecologist after an abnormal cervical smear and the colposcopic examination itself often result in increased anxiety (Markovic-Denic et al., 2017; Marteau et al., 1996). To reduce these pre-colposcopic anxiety levels in patients undergoing colposcopy, previous studies examined the effects of information leaflets, and individually targeted information via telephone or e-mail (Marteau et al., 1996; Bekkers et al., 2002; Howells et al., 1999; De Bie et al., 2011). Galaal et al. reviewed these studies and concluded that these tools could not reduce pre-colposcopic anxiety sufficiently (Galaal et al., 2011). Furthermore, whether these interventions do alter colposcopy consultation time, has never been studied before.

Recently, Ketelaars et al. investigated whether anxiety might be reduced by means of a hospital-dependent non-animation information video. However, they found no significant anxiety reduction compared to women receiving a standard brochure (Ketelaars et al., 2017). They recommended standardising the pre-colposcopy information. In addition, they recommended evaluating the reduction of consultation time. It is important to reduce consultation time and improve colposcopic appraisal, due to the changes in the screening programme for detecting cervical premalignancies in the Netherlands. By incorporating high risk human papillomavirus (hrHPV) PCR testing, referrals are expected to increase with up to 91% (Naber et al., 2016). To our knowledge, anxiety reduction and patient satisfaction have never been studied before using a hospital-independent animation video, which easily can be translated into other languages. Another advantage of a hospital-independent video is that patients will not be influenced by brand awareness of hospitals, which could influence anxiety and satisfaction. Moreover, the impact of an animation video on colposcopy consultation time was unknown.

The objective of this study was to investigate whether supplementing hospital-dependent standard information with a hospital-independent animation video would reduce consultation time in women referred for abnormal cervical cytology. In addition, pre-colposcopy anxiety levels and post-colposcopy satisfaction was measured.

## 2 Methods

### 2.1 Participants and procedure

The study population consisted of women who were referred by their general practitioner to the outpatient clinic of the department of Obstetrics and Gynaecology in one of three participating hospitals in the Netherlands (Catharina Hospital in Eindhoven, Jeroen Bosch Hospital in 's Hertogenbosch and Elisabeth-Tweesteden Hospital in Tilburg) between November 2016 and May 2018. Patients were eligible for inclusion in the current study if they were referred for the first time to the outpatient clinic due to an abnormal cervical smear. Patients were excluded if they have had a colposcopy before or if they were not sufficiently able to understand, speak or read Dutch. All women who matched the selection criteria were informed about the study by telephone and were requested to participate prior to the colposcopy. Women who agreed to participate were not excluded if they did not fully complete the short questionnaire on patient's characteristics. All 122 included participants gave informed consent prior to the study. Ethical approval for this study was granted separately by the medical ethical committee of each hospital.

**Fig. 1:**
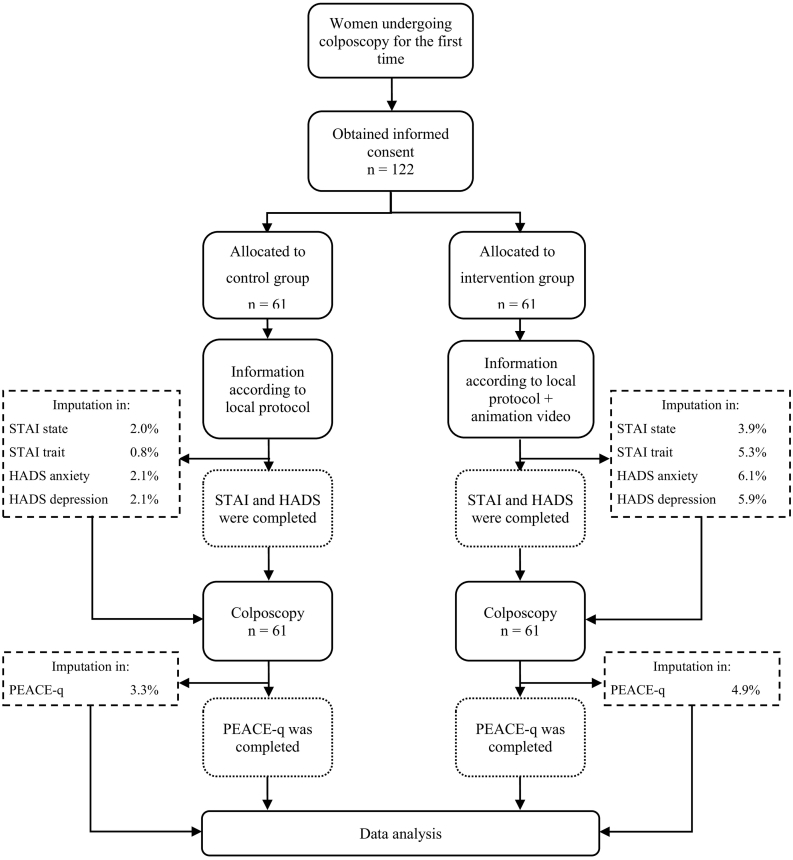
Flowchart: from eligibility to data analysis. Imputation in: percentage of missing data per questionnaire.

Two consecutive cohorts were created (Fig. 1). The first cohort, the control group, consisted of women who received standard written and oral information on colposcopy according to local protocol in one of the three participating hospitals. The second cohort, the intervention group, received the information as mentioned plus an animation video prior to colposcopy.

### 2.2 Sample size calculation

The sample size calculation was based on a prior study performed by De Bie et al. (2011). We hypothesised a difference of 6 in STAI score with a standard deviation of 10 to be significant. Sample size was calculated using power analysis with α = 0.05 (type I error) and β = 0.20 (type II error). It was calculated that 45 eligible women were needed in each group. To allow a dropout rate of 10%, at least 50 women were included to draw statistically valid conclusions.

### 2.3 Animation video

The hospital-independent animation video was designed to explain the different cervical cytology results, the colposcopic procedure, a possible treatment by Large Loop Excision of the Transformation Zone (LLETZ), possible physical effects after the colposcopy and the follow-up procedure. This hospital-independent animation video was created to present standardised information on colposcopy and treatment, which can be used in all clinics performing colposcopy. The video was compiled by gynaecologists of the “Cervix Uteri” working group of the Gynaecological Oncology Centre South (GOCZ) in collaboration with two patients and two academic communication specialists. The video was shown once at the time of the first hospital visit in a separate room. For the reader's convenience, the video has been uploaded at https://vimeo.com/166947180.

### 2.4 Measurements

The consultation time was measured by means of a stopwatch. Prior to colposcopy, all patients completed two questionnaires: the State-Trait Anxiety Inventory (STAI) and the Hospital Anxiety and Depression Scale (HADS), both in Dutch (Van der Ploeg, 1982; Zigmond and Snaith, 1983). In the control group, standard pre-colposcopy and post-colposcopy information was given. In the intervention group, the gynaecologist only provided additional pre- and post-colposcopy information and answered patients' questions after receiving confirmation that the patient had watched the animation video. Immediately after colposcopy, participants were requested to complete the Patient's Experience and Attitude Colposcopy Eindhoven questionnaire (PEACE-q) with questions on: level of being informed, preoccupation, level of stress and level of ease created during consultation (article submitted).

### 2.5 Consultation time

Consultation time was measured with a stopwatch by an observer or by the gynaecologist. Prior to consultation, the gynaecologist was instructed to keep the informative part of the consultation short and was instructed to only answer questions that were raised by the patient. The following components of the consultation were measured: the duration of the conversation prior to the colposcopy, the duration of the colposcopy, and the total consultation time. The three components were measured based on the following protocol. The period of the pre-colposcopy conversation was between closing the door after the patient had entered the examination room and the moment the patient started to undress. The time frame of the colposcopy started when the patient was correctly situated on the examination table and ended when the patient started to dress. The time just before and after the colposcopy examination was not measured as the gynaecologists were instructed to not answer questions during the dressing time. The total consultation time was between closing the door after the patient entered and closing the door again after the patient left. The observer or gynaecologist recorded if there was a reason for extending these time frames due to additional procedures, such as taking a biopsy or LLETZ treatment.

### 2.6 Anxiety and depression

Pre-colposcopy anxiety levels were assessed using the STAI and HADS. The STAI assesses anxiety on 40 items, 20 of which measure the A-state (state anxiety) intensity response scale and 20 of which measure the A-trait (trait anxiety) frequency response scale. The state scale measures anxiety at the time just prior to the colposcopy and the trait scale measures the day-to-day levels of anxiety. All items were assessed using a 4-point Likert scale. The total scores of each response scale range from 20 to 80. Higher scores in both scales indicate a higher level of anxiety. The HADS consists of 14 items: 7 items that assess symptoms of anxiety and 7 items that assess symptoms of depression. All items are scored on a 4-point scale from 0 to 3. The total scores for anxiety and depression range from 0 to 21. A score of 8 or higher in the HADS subscales indicates elevated anxiety and/or depression levels. For anxiety, a score of 8 or more had a sensitivity of 0.9 and a specificity of 0.78, and a score of 8 for depression a sensitivity of 0.83 and a specificity of 0.79 (Bjelland et al., 2002).

### 2.7 Satisfaction

To be able to assess post-colposcopic satisfaction, the PEACE-questionnaire was developed prior to the current study. This questionnaire measures different aspects of satisfaction concerning the patient's perception of the procedure and the patient's perception of the caregiver's attitude before and during the colposcopy. Before developing a first draft scale, women who had recently received colposcopy were invited to participate in a focus group interview. With the permission of the participants, the interview was recorded. The recording was transcribed and sorted by subject. The transcriptions were evaluated by a panel of experts who reached consensus on 15 potential questions for the questionnaire. Next, these 15 questions were answered anonymously by women who had received a colposcopy at the outpatient clinic in one of the three hospitals that also participated in the present study.

A principal component explorative factor analysis of the answers revealed two subscales: the patient's perception of the performed colposcopy procedure and the patient's perception of the caregiver's attitude during the colposcopy procedure. Each subscale comprised 4 items. A confirmative factor analysis on these 8 remaining items revealed an excellent model fit. In the resulting PEACE-questionnaire, the 8 items are rated on a 4-point Likert scale. Higher scores indicate a more positive experience of the colposcopy procedure.

### 2.8 Statistical analysis

For the continuous variables, mean and standard deviation (SD) or median and interquartile range (iqr; 25th and 75th percentile) were given depending on normality. Also depending on normality, missing data were imputed by the mean or median score of each question in the STAI, HADS and PEACE questionnaire. The continuous variables were presented as a percentage and number (n). To assess possible differences in patient characteristics and outcome measures between the control group and the intervention group, Pearson Chi-square was used for categorical variables and Student's *t*-test for continuous variables if normally distributed. Skewed data were analysed with the Mann-Whitney *U* Test. If the expected count was <5 in >20% of the cells, Fisher's Exact test was used for categorical variables. If p ≤ 0.10, differences in patient characteristics were corrected with multivariate linear regression. Statistical analysis was performed using SPSS software (version 25).

## 3 Results

### 3.1 Baseline characteristics

Fig. 1 presents the flow of the study. Due to logistical reasons, the women in the intervention group consisted exclusively of patients who were referred to the Catharina Hospital.

Table 1 shows an overview of the patient characteristics of all 122 included women. No significant differences were found between the two groups in age (p = 0.85), civil status (p = 0.81), educational level (p = 0.23), parity (p = 0.48), reason for cervical smear (p = 0.39) or reason for extension of consultation time (p = 0.07). Fig. 1 presents the percentage of missing data in the control group and intervention group for the different questionnaire subscales.

**Table 1:** Patient characteristics of women with abnormal cervical cytology in the control group and intervention group.

Demographics	Total n = 122		Control group n = 61		Intervention group n = 61		p-Value
	n	%	n	%	n	%	
Age	118		60		58		0.85
20–30 years	50	42.4	27	45.0	23	39.7	
31–40 years	31	26.3	14	23.3	17	29.3	
41–50 years	20	16.9	11	18.3	9	15.5	
51–60 years	14	11.9	6	10.0	8	13.8	
61–70 years	3	2.5	2	3.3	1	1.7	
Civil status	121		61		60		0.81
Married/living together	66	54.5	34	55.7	32	53.3	
Single	29	24.0	15	24.6	14	23.3	
Divorced/split up	15	12.4	8	13.1	7	11.7	
Living apart together (LAT)	11	9.1	4	6.6	7	11.7	
Educational level	121		61		60		0.23
Primary education	1	0.8	1	1.6	0	0.0	
Secondary education	71	58.7	32	52.5	39	65.0	
Tertiary education	49	40.5	28	45.9	21	35.0	
Parity	121		61		60		0.48
Nulliparity	57	47.1	31	50.8	26	43.3	
Primiparity	25	20.7	14	23.0	11	18.3	
Multiparity (2 times)	32	26.4	14	23.0	18	30.0	
Multiparity (>2 times)	7	5.8	2	3.3	5	8.3	
Reason for cervical smear	115		58		57		0.39
National Screening Programme	77	67.0	41	70.7	36	63.2	
Patient's own request	38	33.0	17	29.3	21	36.8	
Reason for extension time	122		61		61		0.07
No	68	55.7	39	63.9	29	47.5	
Yes	54	44.3	22	36.1	32	52.5	

### 3.2 Outcome measures

Table 2 presents the mean (SD) and median (iqr) scores of the STAI, HADS and PEACE questionnaire. No significant differences were observed between the control group and the intervention group in STAI state anxiety (p = 0.16), STAI trait anxiety (p = 0.12), difference between STAI state and trait score (p = 0.81), HADS anxiety (p = 0.58), HADS depression (p = 0.68) and PEACE-q (p = 0.55). The women in the intervention group scored higher on STAI state (mean 44.2 vs. 46.8) and STAI trait (mean 36.0 vs. 38.2) questionnaires, respectively. However, the mean difference between STAI state and trait was 8.2 in the control group and 8.6 in the intervention group. The mean state anxiety score of all the participants was 45.5 (SD 10.5). In addition, we performed an analysis to compare STAI and HADS scores in older and younger women. Women of 40 years of age or younger (n = 81) are compared to women who are 41 years or older (n = 37). No significant differences were found between the older and the younger women.

**Table 2:** Mean and median scores of STAI, HADS and PEACE-q in women with abnormal cervical cytology.

	Total n = 122	Control group n = 61	Intervention group n = 61	p-Value	≤40 years n = 81	>40 years n = 37	p-Value
STAI state Mean (SD)	45.5 (10.5)	44.2 (10.6)	46.8 (10.4)	0.16	46.5 (10.5)	42.9 (9.7)	0.08
STAI trait Mean (SD)	37.1 (7.7)	36.0 (8.5)	38.2 (6.8)	0.12	37.1 (7.7)	36.6 (8.1)	0.76
Difference STAI state and trait Mean (SD)	8.4 (10.3)	8.2 (9.9)	8.6 (10.7)	0.81	9.6	5.5	0.06
HADS anxiety Median (iqr)	4.0 (2.8–5.3)	3.0 (2.5–5.0)	4.0 (2.5–6.0)	0.58	4.0 (3.0–5.0)	3.0 (2.5–6.0)	0.67
HADS depression Median (iqr)	4.0 (2.0–5.0)	4.0 (2.0–5.0)	4.0 (3.0–5.0)	0.68	4.0 (3.0–5.0)	4.2 (2.0–6.5)	0.68
PEACE-q 8 items Mean (SD)	24.0 (2.5)	24.2 (2.3)	23.9 (2.6)	0.55	23.8 (2.5)	24.0 (2.3)	0.73

STAI = State-Trait Anxiety Inventory; HADS = Hospital Anxiety and Depression Scale; PEACE-q = Patient's Experience and Attitude Colposcopy Eindhoven Questionnaire; SD = standard deviation; iqr = interquartile range in 25–75 percentiles; p = p-value (the Netherlands).

Table 3 shows the median and interquartile range of the duration of the pre-colposcopy conversation, the duration of the colposcopy and the total consultation time. A significantly shorter duration of the pre-colposcopy conversation was found in the intervention group than in the control group (who received the local information brochure), 140 s and 269 s respectively (p < 0.001). The median duration of the colposcopy and the total consultation time were not significantly different (p = 0.11) and (p = 0.34), respectively. Corrected for reason for extension of consultation time by means of multivariate linear regression, no statistical significant reduction was found in duration of the colposcopy (p = 0.09) or of the total consultation time (p = 0.57). The time of the pre-colposcopy conversation remained statistical significantly lower in the intervention group when corrected for extension of consultation time.

**Table 3:** Median times of the consultation components in women with abnormal cervical cytology.

	Control group (seconds) n = 61	Intervention group (seconds) n = 61	p-Value uncorrected^a^	p-Value corrected^b^
	Median (iqr)	Median (iqr)		
Duration of the pre-colposcopy conversation	269 (177.50–385.50)	140 (80.00–213)	<0.001	<0.001
Duration of colposcopy	420 (325.50–565.00)	441 (352.00–620.00)	0.11	0.09
Duration of total consultation time	974 (822.00–1239.50)	907 (772.00–1165.00)	0.34	0.57

iqr = interquartile range in 25–75 percentiles.

a Mann-Whitney U Test.

b Linear regression: corrected for reason for extension time (baseline characteristic) (the Netherlands).

## 4 Discussion

The results showed that the anxiety was not reduced and the satisfaction was not increased in the intervention group compared to the control group. However, there was a significant reduction in pre-colposcopy consultation time in the intervention group.

Our findings regarding anxiety reduction are in agreement with those obtained by other studies, none of which found an association between an intervention and anxiety reduction in women referred for colposcopy (Ketelaars et al., 2017; Byrom et al., 2002; Greimel et al., 1997). Only music during the colposcopy significantly reduces anxiety (Chan et al., 2003). Our results are in contrast with the study of Freeman-Wang et al. (2001), who showed a significant reduction in anxiety levels of women who received information via video in addition to the standard leaflet. The informative video used in this study is live action and hospital-dependent, which differs from our animated hospital-independent video.

There are possible explanations for not finding a significant reduction of anxiety. Firstly, due to access to information on the internet, patients are nowadays better informed than at the time of the Freeman-Wang et al. study, which took place at the end of the 20th century. This explanation could be supported by the fact that the mean state of anxiety of our total population is 45.5 (SD 10.5) compared to 51.1 (SD 13.3) in the Freeman-Wang study. The higher anxiety level found by Freeman-Wang may be explained by a lack of available information at that time. However, one could also argument the opposite: unreliable sources could increase anxiety.

The level of satisfaction did not differ between the two groups nor between the three participating hospitals. An explanation could be that nowadays patients tend to ask questions until they are satisfied. Another explanation could be that the ability of the gynaecologists to provide satisfaction is sufficient regardless of the animation video.

We found no previous studies examining interventions to reduce the duration of the colposcopy consult. Our findings do suggest an association between the animation video reducing pre-colposcopy consultation time, which was the most important clinically relevant result of our study. This result may be explained by the fact that gynaecologists do not have to explain the procedure extensively if the patient has already been informed by the animation video. Since the screening programme for detecting cervical premalignancies in the Netherlands has been changed, and an increase of referrals colposcopy is anticipated, it is of the utmost importance to reduce consultation time while maintaining patient satisfaction. It could conceivably be hypothesised that pre-colposcopy time can be reduced without compromising patient satisfaction as there is no significant difference in post-colposcopy satisfaction in our study results. However, total consultation time might not have been reduced due to the fact that timeslots were already allocated. The next intervention we plan to carry out is decreasing total colposcopy time to study whether this influences patient satisfaction and consultation time.

Ketelaars et al. recommended standardisation of the pre-colposcopy information. The animation video is a different medium and could be altered to serve patients who speak another language. For example, the voice over is being translated into Papiamento now. The impact of the animation video in this study was investigated in the Netherlands, where the national language is Dutch, thus the animation video in Dutch could be used nationwide in the Netherlands. As a result, the pre-colposcopy information would be the same all over the country and the majority of the patients would receive the same information. Furthermore, it could be hypothesised that anxiety might be reduced by showing the animation video even earlier than in this study, e.g. immediately after receiving the result from the general practitioner. The information could prevent higher anxiety levels related to the abnormal cytology results before the anxiety sets in.

The present study has several limitations. First, the STAI and HADS questionnaires are used to measure general anxiety and are not specific to colposcopy. The Process Outcome Specific Measure (PSOM) is a tool specifically designed to measure the psychological burden in women with abnormal cytology and could have been used to measure anxiety more precisely (Rothnie et al., 2014). Secondly, the participants in the intervention group were included in only one of the three participating hospitals. Thirdly, the total number of women who were approached was not measured. However, it is our estimation that <5% of the women declined the invitation. Finally, the amount of missing data was higher in the intervention group and could have influenced the final results.

We recommend that future studies evaluate the introduction of the informative video in a primary care setting. We hypothesize that implementing the video immediately after receiving the abnormal cervical cytology result, at the primary care facility or uploaded in the letter of the Dutch Cervical Screening Programme, could reduce anxiety at the very beginning by putting the results into perspective, as also has been suggested by others before (Boada and Parellada, 2017).

## 5 Conclusion

A hospital-independent animation video did significantly reduced pre-colposcopy consultation time but did not reduce anxiety or increase satisfaction in women with abnormal cervical cytology. Further research should focus on the effects of animation video in a primary care setting.

## Declaration of interests

There were no financial or personal relationships that could influence this study.

## Role of funding source

The creation of the animation video and the development of the PEACE-questionnaire were funded by Stichting Volksgezondheid (VGZ). The funding source had no influence in the collection, analysis or interpretation of data, writing the manuscript and the submission of the manuscript.

## Disclosure of source(s) of financial support

Stichting Volksgezondheid (VGZ).

## Publishable conflict of interest

There were no conflicts of interest.
